# A deep learning‐based framework (Co‐ReTr) for auto‐segmentation of non‐small cell‐lung cancer in computed tomography images

**DOI:** 10.1002/acm2.14297

**Published:** 2024-02-19

**Authors:** Tenzin Kunkyab, Zhila Bahrami, Heqing Zhang, Zheng Liu, Derek Hyde

**Affiliations:** ^1^ Department of Computer Science, Mathematics, Physics and Statistics University of British Columbia Okanagan Kelowna British Columbia Canada; ^2^ School of Engineering The University of British Columbia Okanagan Campus Kelowna British Columbia Canada; ^3^ Department of Medical Physics BC Cancer – Kelowna Kelowna Canada

**Keywords:** auto‐segmentation, deep learning models, encoder‐decoder, GTV, non‐small cell‐lung cancer

## Abstract

**Purpose:**

Deep learning‐based auto‐segmentation algorithms can improve clinical workflow by defining accurate regions of interest while reducing manual labor. Over the past decade, convolutional neural networks (CNNs) have become prominent in medical image segmentation applications. However, CNNs have limitations in learning long‐range spatial dependencies due to the locality of the convolutional layers. Transformers were introduced to address this challenge. In transformers with self‐attention mechanism, even the first layer of information processing makes connections between distant image locations. Our paper presents a novel framework that bridges these two unique techniques, CNNs and transformers, to segment the gross tumor volume (GTV) accurately and efficiently in computed tomography (CT) images of non‐small cell‐lung cancer (NSCLC) patients.

**Methods:**

Under this framework, input of multiple resolution images was used with multi‐depth backbones to retain the benefits of high‐resolution and low‐resolution images in the deep learning architecture. Furthermore, a deformable transformer was utilized to learn the long‐range dependency on the extracted features. To reduce computational complexity and to efficiently process multi‐scale, multi‐depth, high‐resolution 3D images, this transformer pays attention to small key positions, which were identified by a self‐attention mechanism. We evaluated the performance of the proposed framework on a NSCLC dataset which contains 563 training images and 113 test images. Our novel deep learning algorithm was benchmarked against five other similar deep learning models.

**Results:**

The experimental results indicate that our proposed framework outperforms other CNN‐based, transformer‐based, and hybrid methods in terms of Dice score (0.92) and Hausdorff Distance (1.33). Therefore, our proposed model could potentially improve the efficiency of auto‐segmentation of early‐stage NSCLC during the clinical workflow. This type of framework may potentially facilitate online adaptive radiotherapy, where an efficient auto‐segmentation workflow is required.

**Conclusions:**

Our deep learning framework, based on CNN and transformer, performs auto‐segmentation efficiently and could potentially assist clinical radiotherapy workflow.

## 1 INTRODUCTION

Lung cancer is the leading cause of cancer‐related mortality in Canada. Approximately 30 000 Canadians were diagnosed with lung cancer in 2022, representing about 13% of all new cancer cases.^1^ In the same year, close to 237 000 people were estimated to have lung cancer diagnosis in the United States of America, which is about 12% of all new cancer cases.^2^ Lung cancer can be broadly divided into two categories: small‐cell‐lung‐carcinoma (SCLC) and non‐small‐cell‐lung‐carcinoma (NSCLC). SCLC represents 15% of all lung cancer cases, whereas 85% of all lung cancer cases are NSCLC.^3^, ^4^

Treatment of lung cancer depends on the stage and as well as the patient's overall health. Radiation therapy is one of the primary treatment techniques which is used for all types and stages of lung cancer.^5^ It is estimated that over half of the lung cancer patients undergo radiation therapy for either palliation or cure of the disease.^6^ Radiation therapy planning relies on the following steps; (1) imaging to define the staging, and to determine if a radiation therapy is a suitable treatment method, (2) delineation of volumes (gross tumor volume [GTV], clinical target volume [CTV], organs at risk [OARs)] etc.) (3) optimizing the placement of radiation beams in a treatment plan to ensure tight dose conformity, and finally (4) follow‐up to assess the clinical outcome.^7^ In radiation therapy, it is necessary to accurately deliver the radiation dose to the tumor, while sparing the organs‐at‐risk (OARs) to minimize radiation‐induced complications.^8^ Consequently, the overall treatment outcome is dependent on an accurate segmentation of the tumor volume. Furthermore, with the use of precision treatments such as stereotactic ablative radiotherapy (SABR) and stereotactic radiosurgery (SRS), accurate delineation of tumor volume is even more important to reduce the chance of a geometric miss. As such, an accurate and efficient segmentation workflow is vital for optimal radiation therapy treatment planning, in order to achieve a high tumor control probability and a low normal tissue complication rate.

The radiation oncologist's manual delineation of the disease on a computed tomography (CT) image, with the help of magnetic resonance imaging (MRI) or positron emission tomography (PET), is generally considered the gold‐standard for segmentation.^9^ Unfortunately manual segmentation is labor‐intensive and can be subject to inter‐ and intra‐ observer variability. Established auto‐segmentation techniques typically use the shallow features of the region of interest (ROI) defined in the medical images, for instance, gray scale and texture information, which usually requires some manual input and is also subjected to user variability.^10^, ^11^, ^12^

More recently, adaptive radiotherapy (ART) has become an active area of research in the radiation therapy community.^13^ The idea behind ART is to address and adapt to the changes in patient anatomy/physiology, including the tumor volumes during the treatment course, thereby, allowing a better targeting of the disease. In contrast to conventional treatment techniques which typically utilize one plan for the whole course of treatment, the ART process involves updating the treatment plan whenever necessary, throughout the course of the treatment.^14^ However, the clinical implementation of the ART workflow has numerous technical challenges, one of which is contouring the OARs and tumor volumes accurately and efficiently for treatment planning. Reducing the time for contouring OARs and tumor volumes will not only be beneficial for the radiation therapy workflow, but may also improve the patient's comfort during the ART treatment process^14^ Therefore, an accurate and efficient deep learning auto‐segmentation algorithm could have a significant impact on advancing the implementation of ART in cancer clinics.

Auto‐segmentation algorithms have other potential applications in the field of radiomics. Radiomics is defined as a quantitative image analysis tool where a large number of mathematical features are extracted from the medical images. These features are used for personalized precision diagnosis and treatment, as well as predicting the clinical outcome.^15^, ^16^ Since radiomics studies utilize a large number of medical images, the segmentation workflow can be time‐consuming and labor‐intensive, which could be aided by an efficient auto‐segmentation algorithm.

Deep learning has the potential to advance the auto‐segmentation field by bringing advantages both in terms of accuracy and efficiency. Deep learning algorithms learn feature representations from medical images and use these deep features to perform segmentation task without any user interventions.^17^ Deep learning auto‐segmentation tasks have been studied in various organ sites such as head and neck, breast, and lung.^7^ In terms of lung cancer, deep learning models have been used to contour OARs in past studies, with relatively high accuracy.^8^ However, only a few studies have segmented tumor volumes for lung cancer using different imaging modalities.^8^, ^18^, ^19^, ^20^ Bi Nan et al. implemented a deep learning model to segment the clinical target volume (CTV) of a lung tumor using the ResNet‐101 algorithm.^21^ Another recent study by Zhang et al. proposed a modified‐ResNet algorithm which outperformed the widely used U‐Net algorithm in segmenting CT images of NSCLC patients, with Dice score of 0.73 (ResNet) and 0.64 (U‐Net).^9^ A study by Wang et al., introduced a convolution neural network (CNN)‐based architecture known as adaptive neural network (A‐net) for automated contour on weekly MRI images.^22^ The deep learning technique employed in the study obtained a Dice score value of 0.82 and precision of 0.81 respectively. ResNet U‐Net was proposed by Zhang et al. to contour GTV of NSCLC patients on CT images.^9^ The Dice score achieved using the proposed network in their study was 0.73, which compared to U‐Net, was 0.64. According to a literature review in 2021, deep learning studies of the segmentation of GTVs in lung resulted in average Dice scores below 0.8.^8^ More recently, Yu et al. proposed 3D ResSE‐Unet, inspired by ResNet, to auto‐segment stage III NSCLC patients with Dice similarity coefficient of 0.74 with average time per patient of 10 s.^19^ Sergey et al. implemented an automatic pipeline that performed detection and volumetric segmentation of NSCLC on CT‐images with median Dice similarity coefficient of 0.82 using multiple datasets. The study summarized that the radiation oncologists favored the proposed auto‐segmentation tool in 56% of the cases.^18^

Convolution neural network (CNN) is a well‐established deep learning tool that is used for medical image segmentation. However, due to the localized receptive field of the convolutional operation, the CNN learning is limited both in terms of global and semantic information.^23^, ^24^, ^25^ The CNN's inability to capture a multi‐scale information leads to inferior image segmentation, particularly when tumor volumes with different sizes and scales are presented.^25^ To overcome the CNN model's constraints associated with capturing global and contextual information, self‐attention mechanisms inspired by human cognitive processes have been developed. The primary purpose of self‐attention is to focus on the crucial data while de‐emphasizing the less relevant information. Furthermore, this selective emphasis of the self‐attention mechanism helps prevent information overload in systems with a limited working memory. Transformers, an attention‐based model, was proposed by Vaswani et al. to obtain the global information and contextual understanding of data with the capability to model long‐range dependencies within them. In the past, Transformers have shown promising results in natural language processing (NLP) and various computer vision tasks.^26^ More recently, Transformers based models have been applied to various image segmentation tasks, achieving accurate segmentation results.^24^ Furthermore, a deformable transformer has been developed to mitigate the issues with long training epochs associated with transformers, as well as low performances in small object detection.^27^

In this study, we propose a novel deep learning architecture based on convolutional neural networks (CNN), residual blocks, and transformers (Co‐ReTr), to accurately auto‐segment the GTV in CT‐images of NSCLC patients.^28^, ^29^ The proposed framework consists of three modules: an encoder, a transformer, and a decoder. Under this framework, CNNs and residual blocks were used to build the encoder and efficiently extract feature representations from the CT images. Furthermore, the deformable transformer is inserted to extract global context information and model long‐range dependency between pixels. There are three important contributions of this work: (1) We explored a novel deep learning architecture using a combination of CNNs, residual blocks, and transformers for 3D medical image segmentation; (2) We introduced an integration of convolutional neural networks and residual blocks to efficiently extract feature representation with fine‐grained details; (3) There are currently no published studies which used this combination of deep layers to auto‐segment GTV on CT images of NSCLC. Moreover, up to the best of our knowledge, there are no prior studies that compared more than five deep learning algorithms to evaluate its performance in segmenting GTV in the same NSCLC dataset of CT images. The goal of the study is to determine the best performing deep learning algorithm, which could be used for a large‐scale radiomics study or improve the workflow for novel radiation treatment planning, such as adaptive radiotherapy.

## 2 MATERIALS AND METHODS

### 2.1 Materials

A total of 676 pre‐treatment CT images of NSCLC patients were used in the current study. The entire dataset comprised of three different sources; (1) NSCLC radiomics dataset^28^, ^29^ from The Cancer Imaging Archive (TCIA); (2) NSCLC radiogenomics dataset from TCIA^30^; (3) Our clinical database (BC Cancer—Kelowna). The first two datasets presented here were downloaded from the TCIA database. One manual contour by a single radiation oncologist was presented for each CT‐image in the training set. The first dataset containing CT images and GTV annotation (NSCLC—Radiomics) was originally obtained from the Maastro clinic in the Netherlands. A helical CT scan with 3 mm slice thickness, with or without contrast, was used for CT‐image acquisition.^31^ The second dataset (NSCLC—radiogenomics) was obtained from both the Stanford University School of Medicine and Palo Alto Veterans Affairs Healthcare System. A scanning parameter of slice thickness (0.625–3 mm, median 1.5 mm), and tube current of 124–699 mA, and 80−140 kVp was used for these CT acquisitions.^32^ Our third dataset was retrospectively collected out of 240 patients from our clinical database, who eventually received stereotactic ablative radiotherapy (SABR) treatment. The patients’ data were exported from our clinical database and anonymized to remove all the patient identifier information. The CT‐images from our cancer center were acquired with 120 kVp, tube current of 79–438 mA, a slice thickness ranging from 1 to 2.5 mm, with majority of the images reconstructed with submillimeter inplane pixel spacing. The images used in our test set were acquired using both Phillips (Phillips, Amsterdam, The Netherlands) and GE Medical Systems (GE Medical System, Chicago Illinois) CT scanners. Sourcing the CT images and segmentations from three datasets, collected from different centers, provided a diverse source of data for a more generalized application.

The first two datasets (*n* = 563) were used for training and validation with random assignments between the two cohorts, and our clinical data (*n* = 113) were used for independent testing of the deep learning models. The training set comprised of NSCLC patients with lung cancer stages ranging from T1a to T4. The test set comprised mainly of early stages NSCLC patients, although a few were diagnosed with later stages (T3 mainly). This is due to the fact that the entire database was built for the NSCLC patients who are eligible for lung SABR treatment. The CT images of those patients without GTV annotations were removed from our database, which originally included 240 patients. The GTV contours within our NSCLC clinical dataset were delineated by multiple radiation oncologists. Table 1 provides the information regarding characteristics of the NSCLC tumors presented in the current study.

**TABLE 1:** Baseline characteristics of the patients in training and test set.

Characteristic	Training test, *n* = 563	Test set, *n* = 113
**Age median (range)**	69 (34–91)	70 (55–80)
**Male: Female**	70%:30%	54%:46%
**Tumor site information**		
Right: Left	250:294	57:66
Tumor volume range (cc)	0.06–653.66	0.18–52.73
Tumor volume median (cc)	16.57	16.44
Tumor volume maximum (cc)	653.66	52.73
Tumor volume minimum (cc)	0.06	0.18
**Tumor type**		
Adenocarcinoma	178	23
Squamous cell carcinoma	191	13
NSCLC (not specified)	75	77
Large cell carcinoma	119	0

Abbreviation: NSCLC, non‐small cell‐lung cancer.

### 2.2 Methods

**FIGURE 1:**
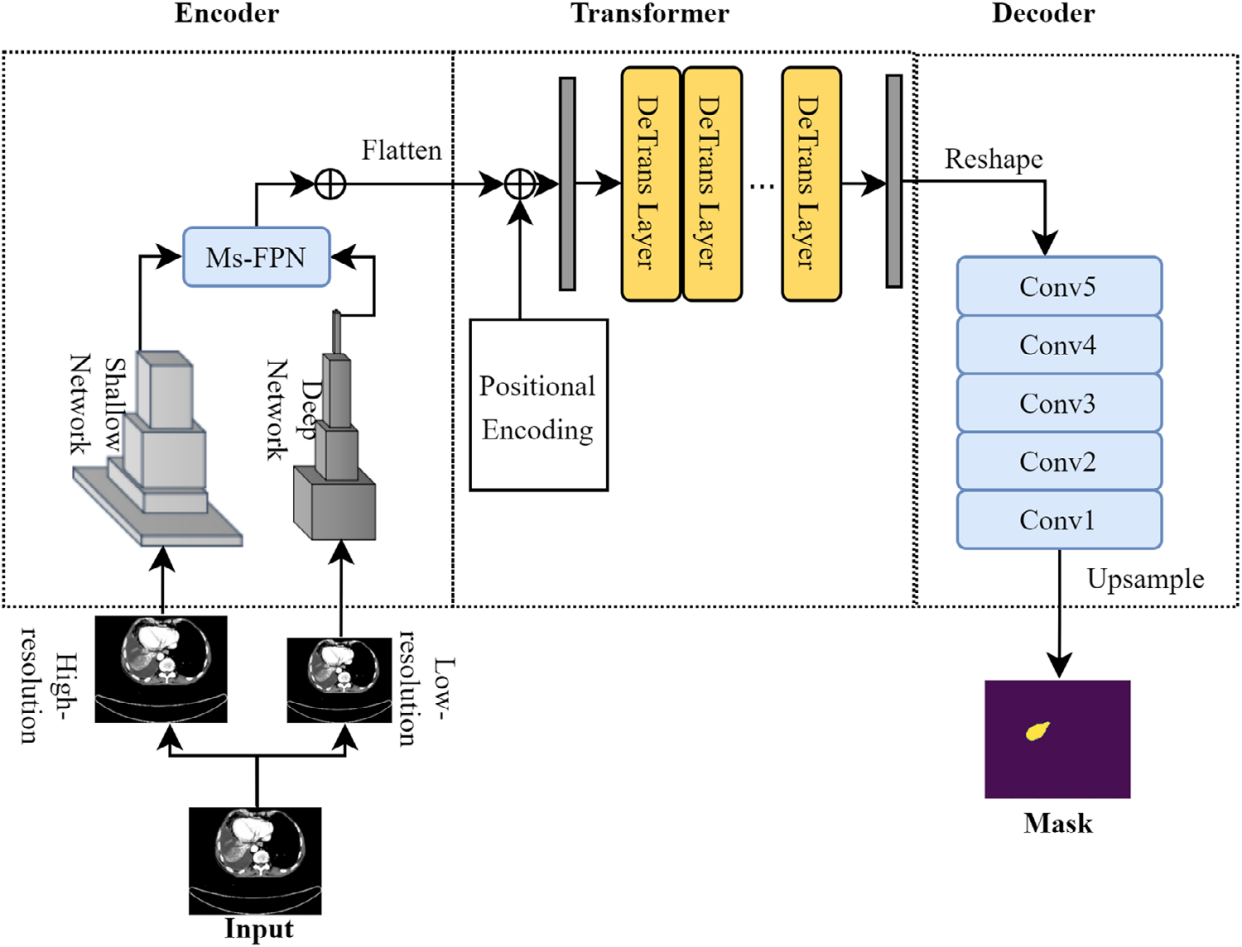
The full architecture of Co‐ReTr consisted of three modules: encoder, transformer, and decoder. In the encoder, input images were passed through a multi‐scale, multi‐depth CNN network to generate super‐resolved feature information of the CT images. Each CNN network contained convolutional blocks and residual blocks to produce a general and fine‐grained detail information. The residual blocks were employed to take low‐level layer information to generate super‐resolved feature maps. In the transformer, several deformable transformer layers were used to extract global information from key sample locations to reduce computational complexity. The decoder was used in the last step to reconstruct the output from the encoded information. CNN, convolutional neural networks; Co‐ReTr, convolutional neural networks, residual blocks, and transformers; CT, computed tomography.

In this section, we present our deep learning framework, Co‐ReTr (Convolutional Neural Networks, Residual Blocks, and Transformers), which was specifically designed to improve the representation of medical images. The integration of CNNs, Residual Blocks, and Transformers collectively forms the backbone of this framework. The comprehensive architecture, as depicted in Figure 1, encompasses three key components: (1) an encoder functioning as a feature extractor; (2) a deformable transformer; (3) a decoder tailored for tasks such as localization and segmentation.

#### 2.2.1 Encoder

**FIGURE 2:**
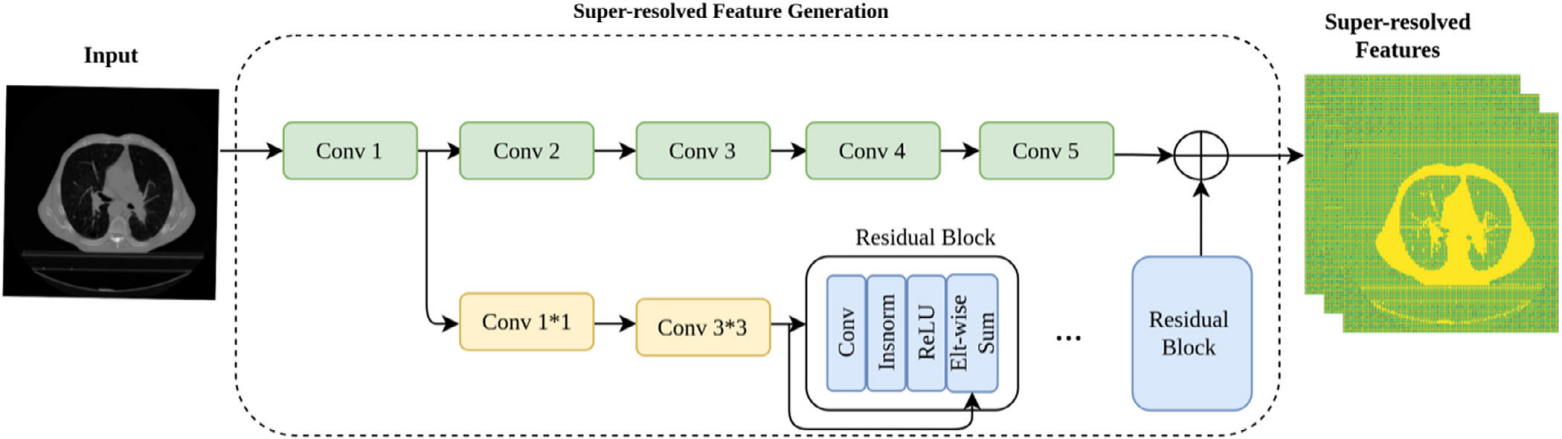
The general structure of shallow and deep convolutional neural networks consisted of 3D convolution blocks to generate general information from input images. The residual blocks were used to generate fine‐grained details which take low‐level information as input and generate super resolved representations. This combination enhances the performance of the model on multi‐scale tumors.

The encoder in our proposed model composed of two CNNs: a deep 3D network and a shallow 3D network, strategically designed to generate diverse features from both high‐resolution and low‐resolution images. To obtain the low‐resolution images, the original input image undergoes a down‐sampling. These low‐resolution images are then processed by the deep CNN to extract semantic information, while the high‐resolution images are fed into the shallow CNN to preserve the positional information and reduce computational complexity. This dual approach of extracting features from both low and high‐resolution images enables our model to accurately segment small tumors while maintaining robust segmentation performance for larger tumors. The shallow CNN architecture consists of five 3D convolutional layers (Conv) interleaved with instance normalization (IN) and Rectified Linear Unit (ReLU), complemented by six stages of 3D residual blocks. Figure 2 provides a visual representation of the structures of both the shallow and deep CNNs. The input image, denoted as X, with dimensions height (*H*), width (*W*), and depth (*D*), is initially processed through the first Conv‐IN‐ReLU block. Fine‐grained details were generated from the low‐level layers of this block. The output of the first convolution layer undergoes a sequence of operations involving a 1 × 1 × 1 convolution filter followed by a 3 × 3 × 3 convolution filter. This process increases the feature dimension and ensures alignment with the last Conv‐IN‐ReLU block. The inclusion of residual blocks in the CNN aids in leveraging low‐level layer information to generate super‐resolved feature maps, as depicted in Figure 2. Each 3D residual block incorporates Conv‐IN‐ReLU units, contributing to the enhancement of pooled features through the application of a 5 × 5 × 5 convolution filter, thereby enriching information from low‐resolution input images.

In the deep CNN, we maintained a similar arrangement of convolutional blocks as in the shallow network, but with an increased number of residual blocks. Specifically, the deep CNN incorporates nine residual blocks compared to the six stages of residual blocks in the shallow CNN. This extension allows the deep network to capture more complex representations and enhances its ability to discern intricate details in medical images.

The feature extractor of the CNNs in our model is designed to generate features that are both multi‐scale and multi‐depth. To effectively handle these multi‐scale hierarchical features, we leveraged a Multi‐Scale Feature Pyramid Network (Ms‐FPN). This specialized network facilitates the propagation of features from high‐level to low‐level and from low‐resolution to high‐resolution, as illustrated in Figure 1.

The operation of the Ms‐FPN can be formulated as follows:

(1) $$F_{i,j}=\left\{\begin{array}{c} Conv_{1\times 1}F_{i,j}+2\times Upsample\;\left(F_{i,j-1}\right)\\ Conv_{1\times 1}F_{i,j}+2\times Upsample\;\left(F_{i,j-1}\right)\\ +\;2\times Upsample\;\left(F_{i+1,j}\right) \end{array}\right.\;$$

where the convolution operation is performed with a 1 × 1 × 1 filter (conv1 × 1 × 1), feature maps denoted as *F*_i,j_ from the CNNs (shallow or deep network) at level *i* and scale *j*, and the up‐sampling operation, which increases the spatial resolution of an image. This operation effectively combines features from both the deep and shallow networks, ensuring a multi‐scale representation that incorporates information from different hierarchical levels. The use of conv1 × 1 × 1 filters helps in managing the dimensionality of the features, and the up‐sampling operation is used to align the features across various resolutions within the multi‐scale feature pyramid network.

#### 2.2.2 Transformer

The encoder proposed in the previous section, while effective in capturing local features, lacks global or contextual information which is crucial to describe the long‐range dependencies between the pixels. This limitation arises because of the intrinsic locality of the convolution operations in both the convolution and residual blocks. Therefore, we have incorporated a deformable transformer and a self‐attention mechanism to capture and model the long‐range contextual information.^33^ The transformer used in our architecture composed of an inputs‐to‐sequence layer and a series of stacked deformable transformer layers. Transformers, originally designed for natural language processing, operate in a sequence‐to‐sequence manner.^26^ In the context of Co‐ReTr, the feature maps generated by the encoder undergo a flattening process to create a 1D sequence. However, flattening the features leads to the loss of crucial spatial information essential for accurate image segmentation.

To mitigate this issue, we introduced a 3D positional encoding sequence for the flattened feature maps, described by the Equations (2) and (3). This positional encoding aims to reintroduce the spatial information into the flattened sequence, allowing the subsequent stages of the network to better understand the spatial relationships among pixels. This strategic addition of 3D positional encoding ensures that the transformer layers can effectively leverage both local and long‐range contextual information for more informed and accurate image segmentation.^26^

(2) $$PoE_{\#}\;\;\left(pos,\;2k\right)=\sin\left(pos\cdot v\right)\;$$

(3) $$PoE_{\#}\;\;\left(pos,\;2k+1\right)=\cos\left(pos\cdot v\right)\;$$

where # ∈ *H*, *W*, *D* denote height, width, depth dimension of the input image and *v* is equal to 1/10000^2k/C/3^. The PoE represents the 3D positional encoding for the feature at position pos, and is calculated independently for each dimension. The sinusoidal and cosine functions introduce variation in the encoding to capture different positional relationships.

The PoE is independently calculated for each dimension (height, width, and depth), resulting in three separate sets of positional encoding sequences. Subsequently, these positional coordinates are concatenated to form a comprehensive positional encoding representation. The concatenated positional coordinates are then combined with the flattened positional encoding, obtained from the initial flattening of the feature maps, using an element‐wise operation. This combined representation serves to shape the input sequence for the transformer, incorporating both spatial and positional information into the sequence for subsequent processing. The integration of this concatenated positional encoding ensures that the transformer can leverage not only the flattened features but also the spatial relationships among pixels, contributing to a more comprehensive understanding of the input image during the sequence‐to‐sequence processing in the transformer layers.

The Deformable Self‐Attention Mechanism, implemented in this work followed the methodology explained by Xie et al., which deviates from the conventional approach where self‐attention mechanism scans feature maps to consider all possible locations around a reference location.^33^ Instead, the methodology adopted in this work aims to enhance efficiency and performance by focusing on a more selective set of key sampling locations. In the typical structure of a transformer, a self‐attention mechanism would attend to all positions in the feature maps relative to a reference location. However, the modification introduced by Xie et al. involves a more targeted approach. Instead of considering all positions, the mechanism focuses on a reduced and strategically chosen set of key sampling locations. This modification serves a dual purpose: it helps reduce the computational burden, thereby improving the overall efficiency, and it is also expected to enhance the performance of the segmentation architecture. By concentrating on a smaller subset of key sampling locations, the deformable self‐attention mechanism becomes more selective in capturing relevant contextual information. This tailored approach allows the model to prioritize crucial areas within the feature maps, potentially leading to more effective feature interactions and contributing to improved segmentation outcomes. The strategic reduction in the number of sampled positions is a trade‐off between computational efficiency and the ability to capture essential long‐range dependencies, creating a more optimized and efficient segmentation architecture.

The DeTrans Layer is a crucial component in the architecture and structure of the model. It is designed to enhance the representation learning process by incorporating a combination of deformable self‐attention, a feed‐forward network, and a layer normalization. The DeTrans Layer is used iteratively by stacking multiple instances of it to create a deformable transformer. This stacking process enables the model to learn hierarchical representations and capture intricate patterns across multiple levels of abstraction. The skip connections in each layer play a crucial role in facilitating the training of a deep network by mitigating the challenges associated with gradient vanishing. The primary components of the DeTrans Layer are summarized below:

1. Deformable self‐attention mechanism: A self‐attention mechanism typically scans the feature maps to find all possible locations around a reference location. However, in this work we followed the methodology explained by Xie et al.,^33^ focusing on a small set of key sampling locations in order to reduce the required time and improve the performance of the segmentation architecture.
2. Feed‐forward network: Following the deformable self‐attention layer, a feed‐forward network is implemented to further process and transform the learned features. This component introduces non‐linearities and enhances the model's capacity to capture complex relationships within the data.
3. Layer normalization: Layer normalization is applied to standardize the activations within each layer. This normalization technique helps stabilize the learning process and improves the convergence of the model during training.
4. Skip connection: To address the issue of gradient vanishing during training, a skip connection is incorporated in each layer of the DeTrans Layer. The skip connection allows the gradients to flow more easily through the network, aiding in the training of deeper architectures.

Overall, the DeTrans Layer combines deformable self‐attention, feed‐forward processing, layer normalization, and skip connections to create an effective building block for the deformable transformer. The stacking of these layers enhances the model's capability to learn and represent complex relationships in the input data, contributing to the overall success of the Co‐ReTr architecture in image segmentation tasks.

#### 2.2.3 Decoder

The transformer in our Co‐ReTr architecture outputs a 1D sequence representing a feature vector. However, reshaping is required to obtain a 3D feature map suitable for subsequent processing. The decoder component of the model completes this task using CNN blocks to up‐sample the feature maps back to the original image resolution. This is achieved using transverse convolutions (also known as deconvolutions or up‐sampling convolutions). Once the up‐sampling is completed, the refined feature map is processed further by residual blocks within the decoder. These residual blocks play a crucial role in generating the fine‐grained information that is necessary for localization and the final segmentation. The inclusion of residual blocks allows our model to capture intricate details and enhance the quality of segmentation outputs. Furthermore, a skip connection is established between the encoder and the decoder. This connection serves to preserve low‐level and detailed information throughout the encoding and decoding stages. By maintaining this connection, the model retains essential features to ensure that low‐level details required for accurate segmentation are not missing. This skip connection is instrumental to achieving a high‐accuracy segmentation, as it facilitates the flow of information between different levels of the network.

#### 2.2.4 System implementation and evaluation

**FIGURE 3:**
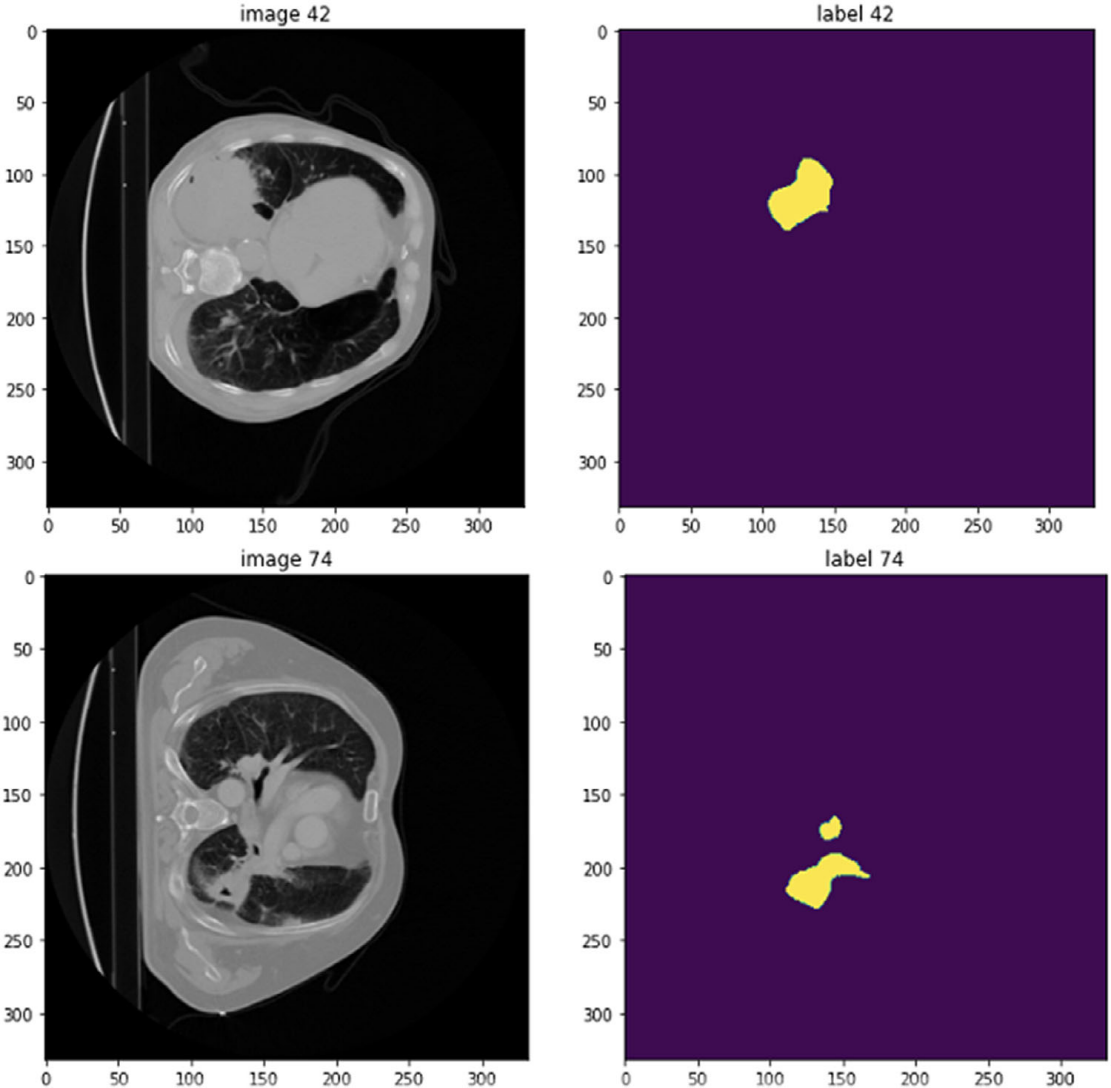
Two samples of training data with corresponding ground‐truth physician contours.

For the preprocessing step, the irrelevant regions were truncated to the range [−1024, 3068] based on the HU values in the CT images, followed by a min‐max normalization. The entire dataset (*n* = 676) is divided into two cohorts: 563 CT images for the training data and 113 CT images for testing. Figure 3 displays two samples of our training data. The test set composed of 113 CT images, which were selected from our clinical database based on the presence of GTV contours and a diagnosis of NSCLC. In the training stage, we randomly cropped sub‐volumes (4 samples) of size 96 × 96 × 96 from CT images as input. In order to avoid over‐fitting, data augmentation including random rotation, scaling, flipping, adding white Gaussian noise, Gaussian blurring, adjusting, brightness and contrast, simulation of low resolution, and Gamma transformation, were used to diversify the training set.^34^ The entire CT data consisted of various slice thickness resolutions, to mitigate this issue, we utilized a resampling technique in SimpleITK (Windowed Sinc Interpolation) to ensure that all slices have uniform thickness. For shallow network, we enhance the resolution of input images by 2, while input images for deep network remain the same size. As the optimizer, the proposed network adopted the stochastic gradient decent method with an initial learning rate of 1e^– 3^. The training batch size was set to 1 and the entire network completed 1000 epochs. All experiments were conducted on a desktop hardware with an Intel Core i7‐8750H CPU, running at 2.20 GHz, and a single GeForce GTX 1070 8GB GPU. However, the training part of the workflow was completed using the cloud computation with higher GPU capability. After training the model, we used our desktop computer with GPU capability for the testing part.

In order to quantitatively assess the performance of the models in this study, two metrics were used, the Dice score and the Hausdorff Distance (HD). The Dice score, also known as the F1 score, is a measure of similarity between two sets. In the context of image segmentation, the Dice score quantifies the overlap between the predicted segmentation mask and the ground truth mask (Equation 4)

(4) $$Dice\;score\;=\;\frac{2\times \left|X\cap Y\right|}{\left|X\right|+\left|Y\right|}$$

where *X* and *Y* are the set of pixels in the predicted mask and ground truth mask, respectively. |·| denotes the cardinality or the size of the set. The Dice score ranges from 0 to 1, where a higher Dice score indicates a superior segmentation performance, with 1 indicating a perfect overlap between the two sets.

The Hausdorff distance (HD) is a metric to quantify the “closeness” of the two sets in terms of their maximum distance. In context of image segmentation, it quantifies the maximum distance between the pixels in a predicted mask to the closest pixel in the ground truth mask. Equation (5) is used to calculate the Hausdorff distance:

(5) $$\begin{array}{ccc} HD\; & = & \;minmax\;\left(g,p\right)=\;\max(maxmin\;\left|\left|g-p\right|\right|,\;\\ & & maxmin\left|\left|g-p\right|\right|)\;equation) \end{array}$$

where *g* and *p* are the pixels of the ground truth and predicted mask respectively. The resulting HD value provides a quantitative measure on how accurately the predicted segmentation mask aligns with the ground truth mask. It also considers the scoring localization similarity by focusing on the boundary delineation. Contrary to the Dice score value, a lower HD value indicates a higher degree of similarity between the two segmentations. This metric is particularly useful when spatial alignment between the two segmentations is of the utmost importance, such as in medical image analysis. Using two evaluation metrics in this study aims to provide a more comprehensive comparison of the performances, in terms of the overlap and spatial congruence of the segmentation masks generated by the different algorithms in relation to the ground truth segmentation.

#### 2.2.5 Comparison with five other deep learning algorithms

Our proposed deep learning algorithm (Co‐ReTr) is compared against five contemporary auto‐segmentation techniques: U‐Net, Att U‐Net, ResNet U‐Net, CoTr, UNETR.^9^, ^25^, ^33^, ^35^, ^36^ The two evaluation metrics (Dice score and HD) were used to quantitatively compare all six of the deep learning methods. Additionally, the inference time, which is the time it takes to contour one single CT‐image, was used to quantify the computational efficiency of each algorithm. Our quantitative analysis is further divided into three sections. In the first section, the Dice score and HD metrics were calculated on the entire test set. Second, we separated the CT‐images with a single tumor and calculated the evaluation metrics. Third, we calculated the same evaluation metrics for a subset of patients with multiple tumors. For the multiple tumors, the deep learning algorithms are required to auto‐segment multiple tumor regions simultaneously. For an unbiased comparison, the models proposed in the previous studies were retrained and the hyperparameters were tuned using our training and validation set, respectively.

## 3 EXPERIMENTAL RESULTS

We conducted a series of experiments to evaluate the overall performance of the proposed deep learning framework in segmenting GTV on our clinical NSCLC patient dataset. We compared our model with five contemporary 3D medical image segmentation techniques (two techniques published previously in NSCLC segmentation): U‐Net, Att U‐Net, ResNet U‐Net, CoTr, UNETR.^9^, ^25^, ^33^, ^35^, ^36^ These deep learning algorithms were trained and tested on a collection of diverse NSCLC data, and their performance was compared against our proposed framework.

The Dice score and the HD metric were used to evaluate the accuracy of the segmentation algorithms. As shown in Table 2, our proposed framework (Co‐ReTr) achieved an overall average Dice score of 0.92, which outperformed the contemporary five auto‐segmentation deep learning models by 3.26%, 9.78%, 15.21%, and 17.39% respectively. With respect to the HD metric, our proposed framework obtained the highest score and outperformed the best competing model by a considerable margin (See Table 2). The capability of learning various features from small to large sized GTVs, along with the long‐range spatial dependencies, has allowed our model to attain the highest accuracy. The convolution‐based frameworks (CoTr, U‐Net, Att U‐Net, ResNet U‐Net) performed well for the larger‐sized GTV segmentation.^9^, ^25^, ^33^, ^35^, ^36^

**TABLE 2:** Quantitative comparisons of segmentation performance of proposed model with five other deep learning techniques on non‐small cell‐lung cancer (NSCLC) dataset.

Models	Dice score	HD	Inference time (s)
U‐Net	0.76	4.097	24.01
Att U‐Net	0.78	4.086	22.19
ResNet U‐Net	0.78	4.021	21.28
CoTr	0.83	3.862	19.21
UNETR	0.89	1.432	12.08
**Co‐ReTr**	**0.92**	**1.333**	**12.03**

Abbreviations: Co‐ReTr, convolutional neural networks, residual blocks, and transformers; HD, Hausdorff Distance.

The NSCLC datasets include patients with single tumor and multiple tumors on their planning CT scans. Further experiments were conducted to evaluate the performances of the proposed framework and other deep learning methods in contouring both single tumor and multiple tumors independently. Table 3 reports the performance of the models in terms of Dice score and HD.

**TABLE 3:** Quantitative comparisons of segmentation performance of proposed model with other techniques on non‐small cell‐lung cancer (NSCLC) dataset.

	One tumor		Multiple tumors	
Models	Dice score	HD	Dice score	HD
U‐Net	0.88	3.021	0.64	5.173
Att U‐Net	0.90	3.006	0.67	5.166
ResNet U‐Net	0.88	2.963	0.66	5.945
UNETR	0.94	1.010	0.84	1.852
Co‐ReTr	0.98	1.003	0.89	1.662

Abbreviations: Co‐ReTr, convolutional neural networks, residual blocks, and transformers; HD, Hausdorff Distance.

**FIGURE 4:**
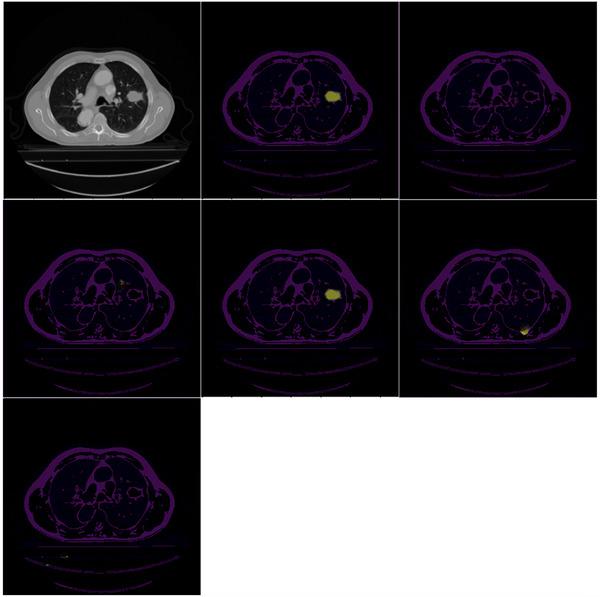
Qualitative comparison of different models on non‐small cell‐lung cancer detection. Top (left: Original image, center: UNETR, right: U‐Net), Middle (left: Co‐Tr, center: Co‐ReTr, right: ResNet Unet), bottom (Att U‐Net). As shown in the figure, our proposed framework visualizes the fine‐grained details of tumors compared to other models based on the two scenarios. For single tumor segmentation, Co‐ReTr outperforms the second‐best technique by 4.08% in terms of Dice score. Regarding the performance on the images with multiple tumors, the proposed framework still outperformed the more recent segmentation techniques both in terms of the Dice score and HD 95% metrics. Co‐ReTr, convolutional neural networks, residual blocks, and transformers; HD, Hausdorff Distance

**FIGURE 5:**
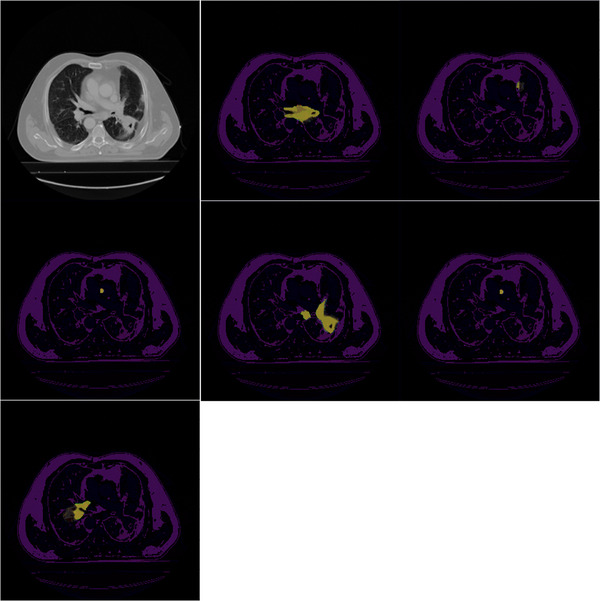
Qualitative comparison of different models on non‐small cell‐lung cancer (NSCLC) detection in the case of two or more tumors. Top (left: Original Image, center: UNETR, right: U‐Net), Middle (left: Co‐Tr, center: Co‐ReTr, right: ResNet U‐Net), Bottom (Att U‐Net).

Table 3 shows that the deep learning models U‐Net, Att U‐Net, ResNet U‐Net, CoTr, and UNETR performed inferior to the Co‐ReTr in terms of the two‐evaluation metrics.^9^, ^25^, ^33^, ^35^, ^36^ Qualitative comparisons between the NSCLC segmentations are presented in Figures 4 and 5. Our model demonstrates better performance in capturing the fine‐grained details of tumors visually.

We evaluated the importance of two Co‐ReTr modules with an ablation study. Our study investigated the effectiveness of the multi‐resolution input image and the transformers module in the Co‐ReTr framework. Table 4 shows a significant improvement, both in terms of Dice score and HD metrics, with the module that feeds in multi‐resolution of the input image. Table 5 shows a significant improvement of both metrics with the transformer module.

**TABLE 4:** The ablation study with and without the multi‐resolution input in the Co‐ReTr deep learning framework.

Model	Dice score	HD
Co‐ReTr without multi‐resolution input	0.85	3.06
Co‐ReTr with multi‐resolution input	0.92	1.33

Abbreviations: Co‐ReTr, convolutional neural networks, residual blocks, and transformers; HD, Hausdorff Distance.

**TABLE 5:** The ablation study with and without the transformers module in the Co‐ReTr deep learning framework.

Model	Dice score	HD
Co‐ReTr without Transformer module	0.77	4.05
Co‐ReTr with Transformer module	0.92	1.33

Abbreviations: Co‐ReTr, convolutional neural networks, residual blocks, and transformers; HD, Hausdorff Distance.

## 4 DISCUSSION

In this study, we developed a deep learning architecture based on convolution layer, residual blocks, and transformers to automatically generate an accurate segmentation. Our proposed framework outperformed five contemporary auto‐segmentation techniques, as quantified by the Dice score and HD. Specific to its deep learning architecture, our model obtains higher segmentation accuracy by capturing local and global information from multi‐resolution CT images. The encoder module assisted the framework in extracting information from small‐scale to large‐scale tumors. The transformer modeled long‐range spatial dependency in detailed representations. It leveraged self‐attention mechanism and focused attention to key positions for long‐range dependency, which reduced the computational complexity. With these advantages, the proposed framework was able to auto‐segment NSCLC accurately and efficiently. The convolution‐based algorithm implemented in the study were able to perform well in large‐size tumor, however, due to the loss of information by the convolution method, small‐size tumors were not segmented well, compared to our deep learning framework. We evaluated multiple published deep learning models, out of which, CoTr and UNETR are two deep learning segmentation techniques that have been published recently, and to the best of our knowledge, there was no study that used those two techniques in segmenting GTV of NSCLC patients on CT images.^25^, ^33^

The ablation study performed in our study highlighted the importance of the multi‐resolution input and transformer modules in the Co‐ReTR framework (Tables 4 and 5). Utilizing a multi‐resolution input image module enabled the deep learning model to discern distinctive features across a wide range of different GTV sizes. We also evaluated the effectiveness of the context feature extraction using transformers. Table 5 presents the effectiveness of incorporating a transformer module in the current framework. Transformer improved the performance of our model because of its ability to capture long‐range dependencies in the CT data, which is essential in medical imaging where the relationship between structures span across the entire image. Furthermore, they can also handle variable‐sized inputs, as presented in our multi‐resolution input images.

In the current study, we used CT images in particular, because most clinics and hospitals use CT images as their primary medical imaging for radiation therapy planning when treating NSCLC patients. The performances of prior deep learning techniques in auto‐segmentation of lung tumor has average dice score of only 0.8, which needs significant improvement in order to implement it widely in the clinical workflow.^8^

Deep learning model proposed here may outperform the existing workflow in clinics in terms of efficiency, and drastically reduce the workload associated with manual contouring. Manual delineation by a physician could take 15 min per patient to contour GTV of NSCLC.^9^ In the current study, our proposed deep learning model took about 12 s per patient. The reduced contouring time may help reduce the physician's workload, allowing them to dedicate more time to other aspects of the planning process. Furthermore, with the advent of adaptive radiotherapy, this auto‐segmentation algorithm could expedite the contouring process and reduce the clinical workload.

One limitation of the current study is that we did not evaluate the inter‐ and intra‐observer variability of the physician contours, as many of the datasets only had one user's contour. However, our CT images from the current study were sourced from three different datasets, from multiple centers and physicians, providing a fairly diverse dataset. Furthermore, within our clinical database we had multiple physicians that contoured the 113 patients Consequently, the Dice scores achieved in this study are very promising for this application. However, it is important to note that our test set only contained early‐stage NSCLC patients (up to T3), which may have influenced the overall results. The relatively high Dice score, compared to the previous research, may be indicative of an overestimation of the performances of the trained networks with this dataset comprised of less difficult examples. Our clinical dataset was not part of the training set due to the fact that our clinical data is restricted to storage on our server and cannot be exported outside our system. Since we used cloud computation for training the model, it would require us to export the CT data outside of our clinical server. Therefore, not being able to distribute our clinical data both in training and testing is an unavoidable limitation of our current study. However, the advantage of the current workflow is that we are using an entirely different cancer center's dataset for testing as an independent test set. Further external evaluation of our proposed model in segmenting GTVs in more advanced NSCLC lung cancer patients, will be beneficial to provide a more comprehensive evaluation and quantify the true performance of our proposed network.

## 5 CONCLUSION

Deep learning auto‐segmentation techniques have tremendous potential to improve the efficiency of an adaptive radiotherapy treatment workflow, by reducing the time associated with manual contouring. In the field of radiomics, auto‐segmentation algorithms can provide great benefits by segmenting the ROI on the CT images without any manual contouring by the physicians. In this study, we presented a deep learning‐based framework for auto‐segmentation of NSCLC from CT images. The proposed framework consisted of three modules, which includes an encoder, a deformable transformer, and a decoder. In the encoder module, multi‐scale multi‐depth feature extractors were used to extract different features from high‐resolution and low‐resolution images. This assisted the model in detecting multi‐scale tumors in CT images. The Co‐ReTr leveraged the transformer capability to learn long‐range spatial dependencies, and to improve the performance of the model in detecting the tumors. The decoder module was employed to upsample the features and provide the final segmentation results. In conclusion, the Co‐ReTr algorithm has demonstrated the potential to effectively and efficiently (an average of 12 s per patient) learn the discriminative features represented in pre‐treatment CT images and auto‐segment the GTV of NSCLC patients successfully based on the two evaluation metrics. The fast and accurate contouring process of our Co‐ReTr algorithm could be beneficial in advancing the adaptive radiotherapy workflow in various clinics.

## AUTHOR CONTRIBUTIONS

**Tenzin Kunkyab**: Clinical data acquisition; methodology; statistical analysis; manuscript writing. **Zhila Bahrami**: Statistical analysis; interpretation; manuscript writing. **Zheng Liu**: Statistical Analysis; committee member; funding acquisition; manuscript revision. **Heqing Zhang**: Clinical data acquisition; undergraduate research assistant. **Derek Hyde**: Conceptualization; funding acquisition; clinical data acquisition; committee member; interpretation; manuscript revision; supervising the author.

## CONFLICT OF INTEREST STATEMENT

The authors declare no conflicts of interest. This work is currently under provisional patent application with invention disclosure file number 2023‐012.

## ETHICS STATEMENT

This study was approved by the UBC Research Ethics Board (REB) Approval number: H18‐00627‐A007.

## Data Availability

Data Availability Statement for this Work: Our own research data are not available at this time. NSCLC Radiomics data is available at [https://doi.org/10.7937/K9/TCIA.2015.PF0M9REI]. NSCLC radiogenomics data is available at [https://doi.org/10.7937/K9/TCIA.2017.7hs46erv].
